# Biofortification of different maize cultivars with zinc, iron and selenium by foliar fertilizer applications

**DOI:** 10.3389/fpls.2023.1144514

**Published:** 2023-09-07

**Authors:** Yan-Fang Xue, Xiao-Jing Li, Wei Yan, Qi Miao, Chun-Yan Zhang, Meng Huang, Jin-Bian Sun, Shi-Jun Qi, Zhao-Hua Ding, Zhen-Ling Cui

**Affiliations:** ^1^National Engineering Research Center of Wheat and Maize, Maize Research Institute, Shandong Academy of Agricultural Sciences, Jinan, China; ^2^Key Laboratory of Biology and Genetic Improvement of Maize in Northern Yellow-Huai Rivers Plain, Ministry of Agriculture, Maize Research Institute, Shandong Academy of Agricultural Sciences, Jinan, China; ^3^College of Agronomy, Liaocheng University, Liaocheng, China; ^4^College of Life Sciences, Shandong Normal University, Jinan, China; ^5^College of Resources and Environment, China Agricultural University, Beijing, China; ^6^Food Crop Cultivation Institute, Linyi Academy of Agricultural Sciences, Linyi, China

**Keywords:** maize, zinc, iron, selenium, bioavailability, ZnO nanoparticles, foliar application

## Abstract

Fertilizer-based biofortification is a strategy for combating worldwide malnutrition of zinc (Zn), iron (Fe) and selenium (Se). Field experiments were conducted to investigate the effects of foliar treatments on concentrations of Zn, Fe, Se, N and bioavailability of Zn and Fe in grains of three maize cultivars grown at three locations. We compared the efficacy of ZnO nanoparticles (ZnO-NPs), Zn complexed chitosan nanoparticles (Zn-CNPs), conventional ZnSO_4_ and a cocktail solution (containing Zn, Fe and Se). All treatments were foliar-applied at rate of 452 mg Zn L^–1^, plus urea. Applying ten-fold less Zn (at rate of 45.2 mg Zn L^–1^) plus urea in the form of ZnO-NPs, Zn-CNPs, or ZnSO_4_ resulted in no increase, or a negligible increase, in grain Zn concentration compared with deionized water. By contrast, among the different Zn sources plus urea applied by foliar sprays, conventional ZnSO_4_ was the most efficient in improving grain Zn concentration. Furthermore, foliar application of a cocktail solution effectively improved grain concentrations of Zn, Fe, Se and N simultaneously, without a grain yield trade-off. For example, the average grain concentrations were simultaneously increased from 13.8 to 22.1 mg kg^–1^ for Zn, from 17.2 to 22.1 mg kg^–1^for Fe, from 21.4 to 413.5 ug kg^–1^ for Se and from 13.8 to 14.7 g kg^–1^ for N by foliar application of a cocktail solution. Because grain yield was significantly negatively correlated with grain nutrient concentrations, the magnitude of increase in grain concentrations of Zn and Fe was most pronounced in the maize cultivar with the lowest grain yield (Zhengdan958 grown in Linyi). Foliar application of a cocktail solution also significantly decreased the phytic acid (PA) concentration, ratios of PA/Fe and PA/Zn in grains, indicating an increased bioavailability of Fe and Zn for human health. In conclusion, we found that a foliar application of a cocktail solution including Zn, Fe, Se and N was most effective for biofortification, but that the grains with the lowest yield contained the greatest concentration of these elements. This finding highlights the need to breed maize varieties that are capable of achieving both high grain yield and high grain nutritional quality to address food security and human health challenges.

## Introduction

Malnutrition of micronutrients including zinc (Zn), iron (Fe), and selenium (Se), commonly known as hidden hunger, is a serious global health problem because these micronutrient deficiencies affect more than one-third of the global population (Welch et al., 2013). Deficiencies of these micronutrients not only impair growth and development owing to their essential roles in metabolism but also negatively affect the immune system of the body against virulent pathogens (Bailey et al., 2015; Read et al., 2019). Insufficient dietary intake of micronutrients and limited dietary diversity are thought to be responsible for human micronutrient deficiencies. These factors are especially important in developing countries, where high proportions of cereal grains with inherently low concentrations of Zn, Fe and Se, such as wheat, rice and maize, are consumed as staple foods (Wang et al., 2013; Beal et al., 2017; Cakmak and Kutman, 2018). The bioavailability of Zn and Fe in cereal grains is also relatively low due to the presence of antinutritional compounds such as phytic acid (PA) and phenolic compounds (Welch and Graham, 2004).

Maize is a major cereal crop and food source for both humans and animals, with a global production of 1210 million tons in 2021. In China, maize production reached 273 million tons in 2021, accounting for 43% of Chinese cereal production and 22.5% of the global maize output (Food and Agriculture Organization of the United Nations (FAO), 2022). It is estimated that maize, as a dietary staple for more than 200 million people, provides around 15% of the world’s protein and 20% of the world’s calories (Nuss and Tanumihardjo, 2010). Micronutrient concentrations in feed and food derived from plants positively affect animal and human health and wellbeing (Grujcic et al., 2018). Therefore, adequate micronutrient concentrations in maize plants are very important for crop productivity and nutritional value.

Among the approaches for biofortification of micronutrient in staple foods, genetic (i.e. plant breeding) and agronomic (i.e. use of fertilizers) approaches are used widely. Fe-biofortified rice, beans, and pearl millet, Zn-biofortified wheat and rice, and provitamin A-biofortified maize, cassava and sweet potatoes have been achieved by conventional breeding strategies (Andersson et al., 2017; Maqbool and Beshir, 2018). Maize, has a much smaller genetic range in grain concentrations of Zn and Fe, limiting the utility of selection-based biofortification (Ortiz-Monasterio et al., 2007; Maqbool and Beshir, 2018). Agronomic biofortification, a fertilizer-based approach, is highly effective in improving grain concentrations of the targeted micronutrients in food crops, as has been demonstrated in wheat, rice, and maize for Zn, Fe, Se and I (Zhang et al., 2010; Wang et al., 2012; Zou et al., 2012; Wang et al., 2013; Mao et al., 2014; Cakmak et al., 2017). These studies also showed that foliar applcations of Zn, I or Se were more effective compared with soil applications for increasing grain concentrations (Wang et al., 2012; Zou et al., 2012; Wang et al., 2013; Cakmak et al., 2017). Compared with foliar appliation of Zn alone, foliar spray of Zn plus nitrogen (N) further improves Zn concentration and Zn bioavailability in wheat grain and flour, indicating a promising strategy for Zn fortification of wheat (Li et al., 2015). Recently, several studies showed that foliar spray of a mixture of micronutrient solution containing simultaneously Zn, Se, I and/or Fe to wheat and rice grown in different countries greatly increased grain concentrations of Zn, Se and I (Zou et al., 2019; Prom-u-thai et al., 2020; Naeem et al., 2022). However, little information is available regarding the effects of foliar spraying a mixture of micronutrients in solution on grain concentrations and bioavailability in maize.

Although foliar application of Zn fertilizer generally had beneficial effects on grain Zn concentration of crops, the effect was dependent on the formulation, the source and the particle size (Palacio-Márquez et al., 2021). The most commonly used Zn foliar sprays are soluble Zn solutions (e.g. ZnSO_4_·7H_2_O and ZnCl_2_) and chelated Zn formulations (e.g. Zn-EDTA) (Montalvo et al., 2016; Veena and Puthur, 2022). Wei et al. (2012) reported that the effectiveness of foliar applied Zn-amino acid and ZnSO_4_·7H_2_O were higher than Zn-EDTA and Zn-citrate for improvement of Zn concentration in rice. Another study revealed that foliar application of ZnSO_4_·7H_2_O alone was more efficient at promoting Zn sorption of cotton plants and citrus leaf compared with ZnSO_4_·7H_2_O combined with EDTA (Stacey and Oosterhuis, 2007). Recently, Zn oxide nanoparticles (ZnO-NPs) have garnered much research interest because nanoparticles are known to be efficiently absorbed, accumulated, and metabolized in plants due to their high surface area to volume ration and less volatilization (Milani et al., 2015). Several studies have shown that, compared to the conventional ZnSO_4_·7H_2_O fertilizers, foliar-applied ZnO-NPs were more effcicent at increasing grain Zn concentration of wheat and maize (Subbaiah et al., 2016; Zhang et al., 2018). In addtion, foliar application of Zn complexed chitosan nanoparticles (Zn-CNPs) resulted in a 26.4% of increase in wheat grain yield and 51.3% increase in wheat grain Zn concentration from 31.0 to 46.9 mg kg^–1^ (Kumar et al., 2021). Furthemore, foliar spray of Zn-CNPs containing the equivalent of 40 mg Zn kg^–1^ could enhance grain Zn concentration in wheat by 36%, similar to the level achieved with 400 mg Zn kg^–1^ applied conventionally an ZnSO_4_·7H_2_O. This comparison highlights the utility of Zn-CNPs as a novel nanofertilizer which enhanced fertilizer use efficiency (Dapkekar et al., 2018). Zn-CNPs have spherical-shaped porous architecture made of cross-linked chitosan sodium tripolyphosphate nanomatrix with -NH_2_ and –OH functional groups to enable encapsulation of Zn ions. The main incentive of encapsulating Zn into a chitosan nanomatrix is to manage the slow release of Zn ions through diffusion *via* dissolution of the chitosan nanomatrix (Choudhary et al., 2019). Therefore, it is necessary to evaluate the effects of foliar application of nano-Zn fertilizers (e.g. ZnO-NPs and Zn-CNPs) on the grain Zn concentration and its bioavailability and to quantify the fertilizer-saving potential in comparison with the conventional ZnSO_4_·7H_2_O under field conditions.

The objective of the present study was therefore to investigate: (i) the effects of foliar spray of a cocktail solution including Zn, Fe, Se and N simultaneously on grain yield and grain concentrations of these nutrients in different maize cultivars grown at different filed locations; (ii) the effects of different forms (conventional ZnSO_4_·7H_2_O, ZnO-NPs, and Zn-CNPs) and doses of foliar Zn fertilizers on grain Zn concentration; and (iii) the effects of foliar treatments on the Fe and Zn bioavailability in maize grain.

## Materials and methods

### Materials

Chitosan (Mol. Wt. 50,000–190,000 Da and 75-85% N-deacetylation) and sodium tripolyphosphate were purchased from Sigma-Aldrich (Shanghai) Trading Co.Ltd. Commercial ZnO NPs were purchased from Shanghai Macklin Biochemical Co., Ltd, with a purity of 99.9% and particle size of 30 ± 10 nm provided by the producer. Zinc sulphate heptahydrate (ZnSO_4_·7H_2_O) and other analytical reagents were purchased from Sinopharm Chemical Regent Co., Ltd, China.

### Zn complexed Chitosan nanoparticles solution preparation

The Zn-CNPs solution was prepared as described by Dapkekar et al. (2018) with some modifications. Briefly, chitosan (3 g) was dissolved in 960 mL of acetic acid (1%, v/v) and the solution was stirred on a magnetic stirrer at ambient temperature (25 ± 3°C) for 1 h and ZnSO_4_·7H_2_O (2 g) was dissolved in chitosan solution. 40 mL of sodium tripolyphosphate (1%, w/v) was then added dropwise to the above solution to form Zn-CNPs solution, which was stirred continuously on magnetic stirrer until there was no precipitation. The content of Zn in this prepared Zn-CNPs solution is 452 mg kg^−1^. In addition, this Zn-CNPs solution was also diluted to 45.2 mg Zn kg^−1^ for foliar Zn treatments.

### Field experiment and experimental design

Field experiments were conducted at three field locations from June to October in 2021 in a winter wheat–summer maize rotation system. The three experimental sites were located at Longshan experimental station of Shandong Academy of Agricultural Sciences in Jinan, Jingzhong experimental station in Zibo and Linyi Academy of Agricultural Sciences in Linyi. The three test sites above are located in Shandong Province, which is one of the major agriculture regions of China and provides around 10% of the national maize production (National Bureau of Statistics of China, 2022). Soil pH (1:2.5 w/v in water), organic carbon, total N, Olsen P, NH_4_OAc-K, DTPA-extractable concentrations of Zn, Fe, Mn and Cu and total Se were shown in **Table 1**.

**Table 1:** Soil properties for the 0–30 cm depth at three experimental locations.

Location	pH	Organic C	Total N	Olsen-P	NH_4_OAc-K	DTPA-Zn	DTPA-Fe	DTPA-Mn	DTPA-Cu	Total Se
		(g kg^-1^)	(g kg^-1^)	(mg kg^-1^)	(mg kg^-1^)	(mg kg^-1^)	(mg kg^-1^)	(mg kg^-1^)	(mg kg^-1^)	(mg kg^-1^)
Jinan (E117°21’11’’, N36°46’42’’)	7.3	9.8	1.10	29.4	116.9	2.26	22.21	16.02	1.29	0.80
Zibo (E118°18’42’’, N36°57’57’’)	7.9	11.0	1.15	15.3	200.8	0.72	11.12	11.43	1.15	0.98
Linyi (E118°27’32’’, N35°11’23’’)	6.2	10.4	1.03	24.5	95.0	1.13	75.84	50.90	2.47	1.13

The experimental design was a split plot design with three replicates. Three maize cultivars (*Zea mays* L.) were planted as the main plots and eight foliar treatments were designated as subplots. Zhengdan958, Denghai605 and Ludan510 (recorded as ZD958, DH605 and LD510, respectively) developed in different years were used in this study. ZD958, released in 1996, represented most widely cultivated new-era cultivar nationally. DH605, released in 2010, represented most widely cultivated new-era cultivar in Shandong Province, China. LD510, newly released in 2021, had high-yielding potential with good performance in rust resistance and lodging resistance. The latter two green-stay hybrids (**Figure S1**) also had the characteristics of dual-purpose grain and silage. Each main plot was designed with an area of 180 m^2^, containing 30 rows (a row spacing of 60 cm) with each row being 10 m long. The average harvest densities were 7.0, 5.7 and 6.2 plants m^–2^ for each cultivar in Jinan, Zibo and Linyi, respectively. In Jinan, 108 kg N ha^–1^ as urea, 60 kg of P_2_O_5_ ha^–1^ as superphosphate and 90 kg of K_2_O ha^–1^ as potassium sulphate were applied before sowing and 72 kg N ha^–1^ as urea was topdressed at 12-leaf stage. Controlled-release fertilizer at the rate of 900 kg ha^–1^ was single applied as basal fertilizer in Zibo (N-P_2_O_5_-K_2_O:26-11-8) and Linyi (N-P_2_O_5_-K_2_O:26-11-11). After the fertilizers were applied, the field plots were immediately irrigated at each location. No obvious water, weed, or pest was observed during the growing season.

Eight foliar treatments were designated as follows: deionized water (control), urea alone (U), urea plus ZnO-NPs at low rate (U+LZnONPs); urea plus Zn-CNPs at low rate (U+LZnCNP), urea plus ZnO-NPs at high rate (U+HZnONPs), urea plus Zn-CNPs at high rate (U+HZnCNP), urea plus ZnSO_4_·7H_2_O (U+Zn), and mixture of urea, ZnSO_4_·7H_2_O, FeSO_4_·7H_2_O and Na_2_SeO_3_ (Cocktail) (**Table 2**). Our preliminary experiment shows that foliar spray of ZnSO_4_·7H_2_O at rate of 2000 mg kg^–1^ (equivalent to 452 mg Zn kg^–1^) is suitable, above which the leaves of maize exhibit obvious toxicity symptoms. Therefore, the different Zn forms were foliar applied at 452 mg Zn kg^–1^ for the latter four treatments. To evaluate the fertilizer-saving potential by use of nano-Zn fertilizers (ZnO-NPs and Zn-CNPs) versus conventional ZnSO_4_·7H_2_O, 10-fold less Zn was foliar applied for treatments U+LZnO and U+LCNP (equivalent to 45.2 mg Zn kg^–1^) (**Table 2**). The Zn content was calculated by considering 22.6% of Zn in ZnSO_4_·7H_2_O and 80% of Zn in ZnO-NPs. For all the foliar treatments, tween 20 as a surfactant (0.01%, w/v) and/or urea (2%, w/v) were freshly added before starting foliar spray. All suspensions were ultrasonicated before leaf application. In order to avoid mutual contamination during foliar treatments, a total of eight rows of maize (with 2 rows of maize spaced between each foliar treatment) were selected and twenty-eight plants grown uniformly from the same row were tagged from each main plots before tasseling stage. The foliar applications were conducted three times every 7–10 days (depending on weather conditions) from tasseling stage on cloudy days or after sunset with windless conditions. 950 L ha^–1^ of each solution was used up at each foliar application for a total of nine split plots at each location.

**Table 2:** Eight foliar treatments including fertilizer types and doses.

Treatment	Types and doses of foliar fertilizer	Pure Zn doses in foliar solution
Control	deionized water	—
U	Urea (2%)	—
U+LZnONPs	Urea (2%) +ZnO-NPs (56.5 mg kg^–1^)	45.2 mg kg^–1^
U+LZnCNP	Urea (2%) +Zn-CNPs	45.2 mg kg^–1^
U+HZnONPs	Urea (2%) +ZnO-NPs (565 mg kg^–1^)	452 mg kg^–1^
U+HZnCNP	Urea (2%) +Zn-CNPs	452 mg kg^–1^
U+Zn	Urea (2%) + ZnSO_4_·7H_2_O (2000 mg kg^–1^)	452 mg kg^–1^
Cocktail	Urea (2%) + ZnSO_4_·7H_2_O (2000 mg kg^–1^) +	452 mg kg^–1^
	FeSO_4_·7H_2_O (2000 mg kg^–1^) + Na_2_SeO_3_ (250 mg kg^–1^)	

### Photosynthetic parameter measurements

Photosynthetic parameters including net photosynthetic rate (P_n_), transpiration rate (T_r_), stomatal conductance (g_s_), and intercellular CO_2_ concentration (C_i_) were measured using a portable Li-6800 photosynthesis system (LI-COR Inc., Lincoln, NE, United States). The ear leaves of plants at 30 and 45 days after silking (DAS) were selected for measurement between 09:00 and 11:00. The photosynthetically active radiation, temperature, and CO_2_ concentration during measurement collection were set at 1200 μmolm^−2^ s^−1^, 25 °C and 400 μmol mol^−1^, respectively. During each measurement, three leaves were taken from each of three replicates of each cultivar grown only in Jinan with four selected foliar treatments including U+HZnONPs, U+HZnCNP, U+Zn and control treatment.

### Chemical analysis

At maturity, twenty spikes treated with foliar application in one row of each split plot were harvested to determine the grain yield (at 15.5% moisture). Sub-samples of three-plant samples were collected and divided into grain and straw. All samples were rapidly washed with deionized water and then oven-dried at 70 °C to determine the dry weight. The dried samples were ground with a stainless steel grinder and then digested using an acid mixture of HNO_3_–H_2_O_2_ in a microwave accelerated reaction system (CEM Corp., Matthews, NC, United States). The concentrations of Zn, Fe, manganese (Mn), copper (Cu), calcium (Ca), magnesium (Mg), phosphorus (P), and K (potassium) in the digested solutions were determined by inductively coupled plasma optical emission spectrometry (ICP-OES, Avio™ 200, PerkinElmer, Waltham, MA, United States). Selenium and boron (B) in the digested solutions were determined by inductively coupled plasma optical emission spectrometry (ICP-MS, NexION^®^ 1000, PerkinElmer, Waltham, MA, United States). Two blanks and a certified reference material (IPE556 grain or IPE883 straw, Wageningen University, the Netherlands) were included in each batch of digestion to ensure analytical quality. Nitrogen concentration in grain samples was determined using a CN analyzer (vario Macro cube, Elementar, Hanau, Germany). Phytate P concentration was analyzed according to the method of Haug and Lantzsch (1983). Phytate P was converted to PA by dividing by 0.282. A tri-variate model that considers Zn and PA in diet, as well as Zn homeostasis in the human intestine has been developed widely used to predict total daily absorbed Zn (TAZ) (Miller et al., 2007). The TAZ is based on reference adults who consume maize flour (300 g day^–1^) as a sole source of Zn and PA and is termed “estimated Zn bioavailability”. The molar ratios of PA to Fe or Zn are often used as indicators for the bioavailability of these minerals (Ryan et al., 2008).

### Statistical analysis

The significance of the effects of the experimental locations, maize cultivars and foliar treatments as well as their double and triple interactions on the reported parameters was evaluated by analysis of variance (ANOVA) using SAS software (SAS 8.0, SAS Institute, Cary, NC, USA). Significant differences between treatment means were determined using Fisher’s protected least significant difference (LSD) test at the 5% level (*P* < 0.05). The relationships among all the investigated parameters were determined by Pearson’s correlation analysis performed by OriginPro 2021. Principal component analysis (PCA) was performed using RStudio to compare the effects of experimental locations, maize cultivars and foliar treatments on various parameters investigated.

## Results

### Grain yield

**Figure 1:**
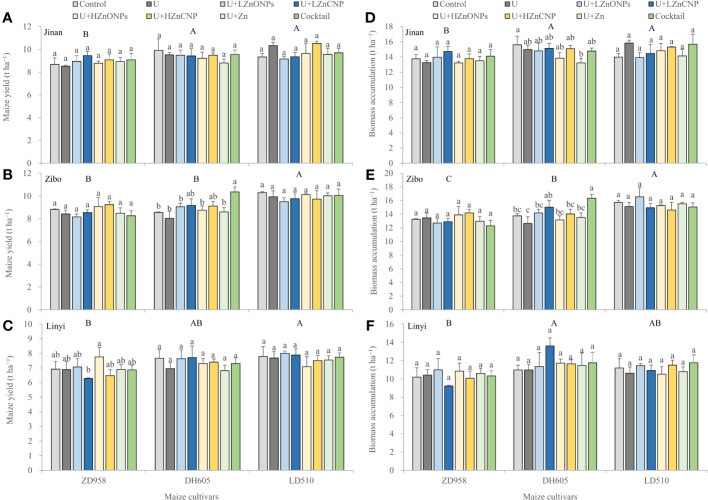
Effects of foliar treatments on grain yield **(A-C)** and biomass accumulation **(D-F)** of different maize cultivars grown in Jinan, Zibo and Linyi, respectively. Error bars represent the standard error of the mean (n = 3). Bars with different lowercase letters represent significant differences among the different foliar treatments within each maize cultivar; bars with different uppercase letters represent significant differences between different maize cultivars (*P* < 0.05). Control: deionized water; U: urea alone; U+LZnONPs: urea plus ZnO-NPs at low rate; U+LZnCNP: urea plus Zn-CNPs at low rate; U+HZnONPs: urea plus ZnO-NPs at high rate; U+HZnCNP: urea plus Zn-CNPs at high rate; U+Zn: urea plus ZnSO_4_·7H_2_O; Cocktail: mixture of urea, ZnSO_4_·7H_2_O, FeSO_4_·7H_2_O and Na_2_SeO_3_.

Three-way ANOVA revealed that grain yield and biomass were first significantly affected by experimental locations, followed by maize cultivars, but not foliar treatments and their double and triple interactions with an exception of a significant effect of the interaction between locations and cultivars on the biomass accumulation (**Supplementary Table 1**). **Figure 1** showed that grain yield gradually increased in the order ZD958<DH605<LD510 at each experimental location. The average grain yield of LD510 and DH605 was 11.6% and 5.2% of higher than ZD958, respectively. Across the three locations, average grain yield in Jinan and Zibo was 28.2% and 25.6% of higher than in Linyi, respectively. However, compared with the control treatment, other foliar treatment generally had less effect on grain yield (**Figures 1A-C**). Similar results were also found for biomass accumulation (**Figures 1D-F**).

### Leaf photosynthetic parameters

**Figure 2:**
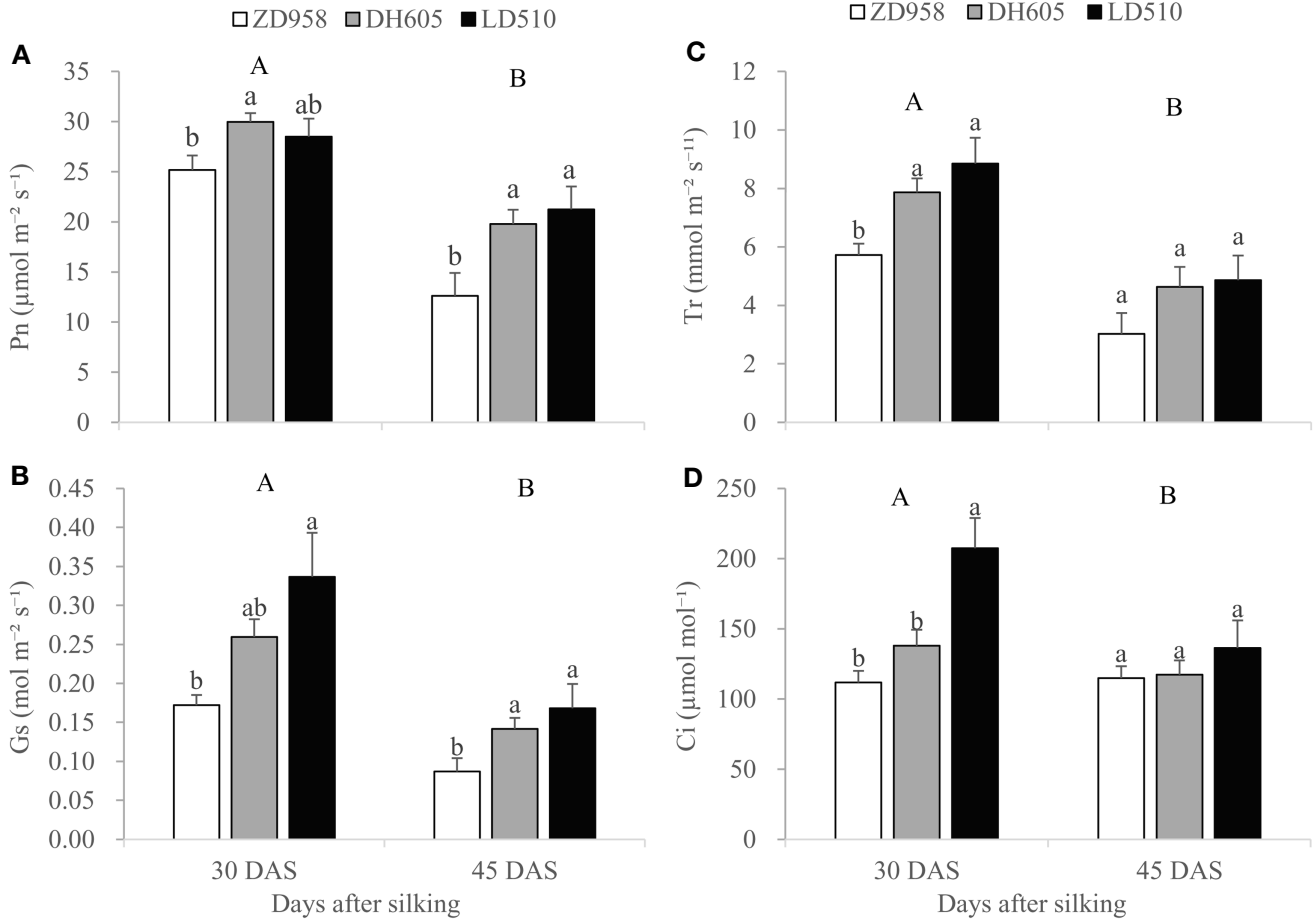
Photosynthetic parameters including P_n_**(A)**, G_s_ **(B)**, T_r_ **(C)**, and C_i_ **(D)** of ear leaves of three maize cultivars at 30 and 45 days after silking (DAS) grown in Jinan. Error bars represent the standard error of the mean (n = 3). Bars with different lowercase letters represent significant differences among the different maize cultivars within each growth stage; bars with different uppercase letters represent significant differences between different growth stages (*P* < 0.05). Pn, photosynthetic rate; Gs, conductance to H_2_O; Tr, transpiration rate; Ci, intercellular CO_2_ concentration.

Two-way ANOVA revealed significant differences between maize cultivars grown in Jinan in the leaf Pn, Gs, Tr and Ci values, but no significant effects of foliar treatments and the interaction between maize cultivars and foliar treatments on these photosynthetic parameters at 30 DAS and 45 DAS (**Supplementary Table 2**). Therefore, data from different foliar treatments were pooled for each maize cultivar for further statistical analysis. **Figure 2** showed that DH605 had significantly higher leaf Pn compared with ZD958 while LD510 had an immediate value at 30 DAS. In addition, LD510 had significantly higher leaf Gs, Tr and Ci values compared with ZD958 while DH605 had immediate values at 30 DAS. At 45 DAS, LD510 and DH605 had significantly higher Pn and Gs compared with ZD958 while there was no significant different between LD510 and DH605. There were also no significant differences in Tr and Ci values among the three cultivars at 45 DAS. With the advance of maize growth, leaf photosynthetic parameters including Pn, Gs, Tr and Ci decreased significantly at 45 DAS compared with at 30 DAS.

### Grain Zn concentration

**Figure 3:**
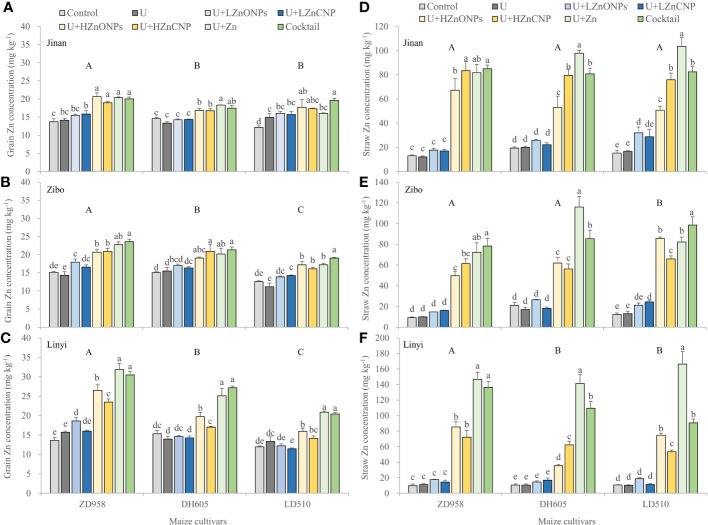
Effects of foliar treatments on Zn concentrations of grain **(A-C)** and straw **(D-F)** of different maize cultivars grown in Jinan, Zibo and Linyi, respectively. Error bars represent the standard error of the mean (n = 3). Bars with different lowercase letters represent significant differences among the different foliar treatments within each maize cultivar; bars with different uppercase letters represent significant differences between different maize cultivars (*P* < 0.05). Control: deionized water; U: urea alone; U+LZnONPs: urea plus ZnO-NPs at low rate; U+LZnCNP: urea plus Zn-CNPs at low rate; U+HZnONPs: urea plus ZnO-NPs at high rate; U+HZnCNP: urea plus Zn-CNPs at high rate; U+Zn: urea plus ZnSO_4_·7H_2_O; Cocktail: mixture of urea, ZnSO_4_·7H_2_O, FeSO_4_·7H_2_O and Na_2_SeO_3_.

As shown in **Figures 3A-C**, grain Zn concentration ranged from 11.2 to 31.8 mg kg^–1^. Irrespective of maize cultivars and locations, compared with control treatment, treatment U did not significantly increased grain Zn concentration with an exception of a significant increase in LD510 grown in Jinan. Treatments U+LZnONPs and U+LZnCNP did not also significantly or slightly increased grain Zn concentration compared with control. However, grain Zn concentration was substantially increased by 36.6–134.0% for ZD958, 11.1–77.1% for DH605 and 18.5–74.9% for LD510 by treatments U+HZnONPs, U+HZnCNP, U+Zn and cocktail compared with control across the three locations. Among the three maize cultivars, the average grain Zn concentration increased in the order LD510<DH605<ZD958. Across the three locations, the average grain Zn concentration increased in the order Jinan<Zibo<Linyi. With high foliar Zn application rate, treatments U+Zn and especially cocktail generally resulted in the highest grain Zn concentration, followed by U+HZnONPs and then U+HZnCNP compared with control (**Figures 3A-C**). Totally, treatments U+Zn and cocktail resulted in the most obvious improvement in grain Zn concentration of ZD958 grown in Linyi. For example, in Linyi, grain Zn concentration was substantially increased from 13.6 to 31.2 mg kg^–1^ for ZD958, from 15.3 to 26.1 mg kg^–1^ for DH605 and from 11.9 to 20.6 mg kg^–1^ for LD510 by treatments U+Zn and cocktail compared with the control treatment, respectively.

### Grain concentrations of Fe, Se, N and PA

Grain Fe concentration ranged from 14.4 to 33.1 mg kg^–1^ (**Figure S2**), grain Se concentration ranged from 11.5 to 717.6 ug kg^–1^ (**Figure S3**) and grain N concentration ranged from 12.4 to 16.6 g kg^–1^ (**Figure S4**), all of which were significantly affected by locations, cultivars, foliar treatments and their double and triple interactions (**Table 3**). Across the three locations, the average concentrations of grain Fe, Se and N consistently and significantly increased in the order Jinan<Zibo<Linyi. However, grain PA concentration was significantly lower in Zibo than in Jinan and Linyi. Among the three maize cultivars, the average grain Fe concentration significantly increased in the order LD510<DH605<ZD958. Grain Se concentration of LD510 was significantly lower than that of DH605 and ZD958. There was no significant difference between DH605 and ZD958 in grain Se concentration. Average grain concentrations of N and PA significantly increased in the order ZD958<LD510<DH605 (**Table 3**). For the foliar treatments, grain Fe concentration was not significantly affected by treatment U and U+LZnCNP, and was increased by 6.4% by treatment U+LZnONPs, increased by around 15% by treatments U+HZnONPs and U+HZnCNP, substantially increased by 26.2% by treatment U+Zn and 29.4% by treatment cocktail, compared with the control. Grain Se concentration was substantially increased from a mean value of 21.4 ug kg^–1^ with control treatment to 413.5 ug kg^–1^ with cocktail while it was not significantly affected by the other foliar treatments (**Table 3**, **Figure S3**). Compared with the control, grain N concentration was significantly increased by 4.4–7.1% by the other seven foliar treatments. However, grain PA concentration was significantly decreased by 6.3–10.6% by the latter four foliar treatments (U+HZnONPs, U+HZnCNP, U+Zn and cocktail) and was not significantly affected by treatments U, U+LZnONPs and U+LZnCNP compared with the control (**Table 3**). In addition, eight foliar treatments did not significantly affect grain concentrations of Mn, Cu, B, K, Mg and Ca (**Supplementary Table 3**).

**Table 3:** Nutrient concentrations in grain (Fe, Se, N, P and PA), straw and shoot (Fe, Se and P) of three maize cultivars with foliar treatments at three experimental locations.

Treatment	Grain nutrient concentration					Straw nutrient concentration			Shoot nutrient concentration		
	Fe	Se	N	P	PA	Fe	Se	P	Fe	Se	P
	(mg kg^–1^)	(ug kg^–1^)	(g kg^–1^)	(g kg^–1^)	(g kg^–1^)	(mg kg^–1^)	(ug kg^–1^)	(g kg^–1^)	(mg kg^–1^)	(ug kg^–1^)	(g kg^–1^)
Location (L)											
Jinan	17.37c	49.79c	14.15c	2.43b	6.71a	192.16a	176.72b	0.71b	96.4a	107.1b	1.66b
Zibo	18.83b	71.77b	14.42b	2.37c	6.01b	120.11c	230.35a	0.55c	64.7c	143.4a	1.542c
Linyi	21.53a	86.56a	15.04a	2.83a	6.67a	157.70b	213.64a	0.99a	81.3b	143.1a	2.03a
Cultivar (C)											
ZD958	21.32a	71.14a	13.96c	2.39c	6.29b	168.21a	212.50ab	0.74a	86.7a	133.9a	1.66b
DH605	19.85b	81.02a	15.22a	2.64a	6.66a	165.77a	222.24a	0.76a	87.0a	145.9a	1.78a
LD510	16.57c	55.97b	14.43b	2.59b	6.45ab	135.99b	185.96b	0.75a	68.8b	113.8b	1.79a
Foliar application (F)											
Control	17.17d	21.39b	13.84b	2.60a	6.73ab	145.55b	91.78b	0.84a	74.2bc	52.6b	1.83a
U	17.31d	21.44b	14.63a	2.56ab	6.89a	148.56b	81.21b	0.79ab	76.1b	48.3b	1.77bc
U+LZnONPs	18.27c	19.85b	14.83a	2.60a	6.42bc	129.86c	79.95b	0.78abc	68.6c	47.5b	1.78ab
U+LZnCNP	17.84dc	18.78b	14.44a	2.61a	6.84a	145.98b	99.62b	0.75bcd	76.1b	55.7b	1.76bc
U+HZnONPs	19.74b	20.23b	14.61a	2.49bc	6.31c	147.75b	91.83b	0.71dc	76.0b	52.1b	1.71d
U+HZnCNP	19.75b	21.50b	14.54a	2.47c	6.26c	154.97b	98.18b	0.69d	80.4b	55.7b	1.67d
U+Zn	21.67a	18.35b	14.66a	2.48c	6.02c	140.82bc	96.57b	0.71bcd	74.6bc	53.7b	1.69d
Cocktail	22.21a	413.46a	14.73a	2.54abc	6.26c	239.79a	1016.07a	0.73bcd	120.4a	683.9a	1.72dc
Source of variation											
L	<0.0001	<0.0001	<0.0001	<0.0001	<0.0001	<0.0001	0.0017	<0.0001	<0.0001	<0.0001	<0.0001
C	<0.0001	<0.0001	<0.0001	<0.0001	0.0179	<0.0001	0.0470	ns	<0.0001	0.0018	<0.0001
F	<0.0001	<0.0001	<0.0001	0.00	0.0001	<0.0001	<0.0001	0.0023	<0.0001	<0.0001	<0.0001
L*C	<0.0001	<0.0001	<0.0001	<0.0001	<0.0001	<0.0001	0.0142	0.001	<0.0001	0.0003	<0.0001
L*F	<0.0001	<0.0001	0.0017	0.01	ns	ns	0.0474	ns	0.0025	<0.0001	<0.0001
C*F	<0.0001	<0.0001	0.0102	ns	ns	0.0023	0.0018	ns	0.0207	<0.0001	ns
L*C*F	0.0018	<0.0001	0.0498	0.00	ns	0.0051	<0.0001	0.0143	0.0182	<0.0001	<0.0001

Means in a column followed by different lowercase letters are significantly different among different locations, cultivars and foliar treatments (P<0.05).

ns indicates nonsignificance. Control: deionized water; U: urea alone; U+LZnONPs: urea plus ZnO-NPs at low rate; U+LZnCNP: urea plus Zn-CNPs at low rate; U+HZnONPs: urea plus ZnO-NPs at high rate; U+HZnCNP: urea plus Zn-CNPs at high rate; U+Zn: urea plus ZnSO_4_·7H_2_O; Cocktail: mixture of urea, ZnSO_4_·7H_2_O, FeSO_4_·7H_2_O and Na_2_SeO_3_.

### Zn, Fe, Se and P concentrations in straw and shoot

Straw Zn concentration ranged from 9.2 to 166.3 mg kg^–1^ (**Figures 3D-F**), straw Fe concentration ranged from 79.1 to 347.9 mg kg^–1^ (**Figure S2**), and straw Se concentration ranged from 37.0 to 1512.1 ug kg^–1^ (**Figure S3**). Irrespective of locations and maize cultivars, treatments U, U+LZnONPs and U+LZnCNP did not significantly increase straw Zn concentration with exceptions of slight increases in LD510 grown in Jinan with treatment U+LZnONPs and grown in Zibo with treatment U+LZnCNP compared with the control. However, compared with the control, the latter four foliar treatments with high Zn spray rates (U+HZnONPs, U+HZnCNP, U+Zn and cocktail) substantially increased straw Zn concentration by 4.2–14.1-fold, 1.7–12.3-fold and 2.3–14.6-fold corresponding to ZD958, DH605 and LD510 among the three locations, respectively, with treatment U+Zn resulting in the greatest increase in straw Zn concentration (**Figures 3D-F**). Compared with the control, only treatment cocktail substantially increased the straw concentrations of Fe by 0.6-fold and Se by 10.1-fold. However, there was no significant difference in the straw concentrations of Fe and Se among the foliar treatments excluding treatment cocktail with an exception of a significant decrease in straw Fe concentration with treatment U+LZnONPs (**Table 3**). Similar results were also found for shoot concentrations of Fe and Se (**Table 3**). Foliar treatments generally decreased P concentration in straw and especially in shoot compared with the control (**Table 3**).

### Molar ratios of PA/Zn and PA/Fe in grain and P/Zn and P/Fe in straw

As shown in **Table 4**, among the three locations, the average molar ratios of PA/Zn and PA/Fe generally increased in the order Zibo<Linyi<Jinan. Among the three cultivars, the average molar ratios of PA/Zn and PA/Fe gradually increased in the order ZD958<DH605<LD510. For foliar treatments, compared with the control and treatment U, the other foliar treatments generally resulted in a significant decrease in the molar ratio of PA/Zn and PA/Fe in grain. For the ratios of P/Zn in straw, compared with the control, the other foliar treatments significantly decreased the molar ratio of P/Zn in straw by 8.6–90.7% with the treatment U+Zn resulting in the greatest magnitude of decrease. For the molar ratio of P/Fe in straw, there was no significant difference among the four foliar treatments including control, U, U+LZnONPs and U+Zn. Compared with the control, the molar ratio of P/Fe in straw was significantly decreased by 12.2–25.6% by the treatments U+LZnCNP, U+HZnONPs and U+HZnCNP and substantially decreased by 50.5% by the treatment cocktail (**Table 4**).

**Table 4:** Molar ratios of PA/Zn and PA/Fe in grain and molar ratios of P/Zn and P/Fe in straw and shoot of three maize cultivars with foliar treatments at three experimental locations.

Treatment	Grain		Straw		Shoot	
	PA/Zn	PA/Fe	P/Zn	P/Fe	P/Zn	P/Fe
Location (L)						
Jinan	41.28a	33.43a	52.6b	7.0c	146.5b	33.0c
Zibo	35.42c	27.55b	49.7b	9.1b	144.6b	45.8b
Linyi	39.43b	27.95b	101.4a	12.5a	206.0a	49.3a
Cultivar (C)						
ZD958	34.07c	26.16c	74.3a	8.6b	159.4b	37.7c
DH605	39.33b	29.40b	63.2b	9.2b	161.9b	40.3b
LD510	42.73a	33.39a	66.2b	10.9a	175.8a	50.1a
Foliar application (F)						
Control	48.61a	34.04a	148.0a	11.2ab	289.6a	48.4ab
U	48.67a	34.04a	135.3b	10.5abc	276.6b	45.1bc
U+LZnONPs	41.40c	30.25b	86.5c	11.7a	213.9d	49.8a
U+LZnCNP	45.88b	33.06a	95.0c	9.8c	231.3c	44.5c
U+HZnONPs	33.03d	27.95c	26.6d	9.4dc	99.1e	43.6c
U+HZnCNP	34.51d	27.84c	21.9de	8.3d	88.7e	39.6d
U+Zn	28.84e	24.84d	13.8e	10.0bc	59.6f	43.7c
Cocktail	28.75e	25.17d	16.2e	5.5e	67.0f	26.9e
Source of variation						
L	<0.0001	<0.0001	<0.0001	<0.0001	<0.0001	<0.0001
C	<0.0001	<0.0001	<0.0008	<0.0001	<0.0001	<0.0001
F	<0.0001	<0.0001	<0.0001	<0.0001	<0.0001	<0.0001
L*C	<0.0001	<0.0001	<0.0001	<0.0001	<0.0001	0.0003
L*F	<0.0001	0.0055	<0.0001	0.0244	<0.0001	<0.0001
C*F	0.0012	0.0008	<0.0001	0.0013	<0.0001	0.0047
L*C*F	0.0205	ns	0.0056	0.0015	0.0002	0.0023

Means in a column followed by different lowercase letters are significantly different among different locations, cultivars and foliar treatments (P<0.05). ns indicates nonsignificance.

Control: deionized water; U: urea alone; U+LZnONPs: urea plus ZnO-NPs at low rate; U+LZnCNP: urea plus Zn-CNPs at low rate; U+HZnONPs: urea plus ZnO-NPs at high rate; U+HZnCNP: urea plus Zn-CNPs at high rate; U+Zn: urea plus ZnSO_4_·7H_2_O; Cocktail: mixture of urea, ZnSO_4_·7H_2_O, FeSO_4_·7H_2_O and Na_2_SeO_3_.

### Total daily absorbed Zn

**Figure 4:**
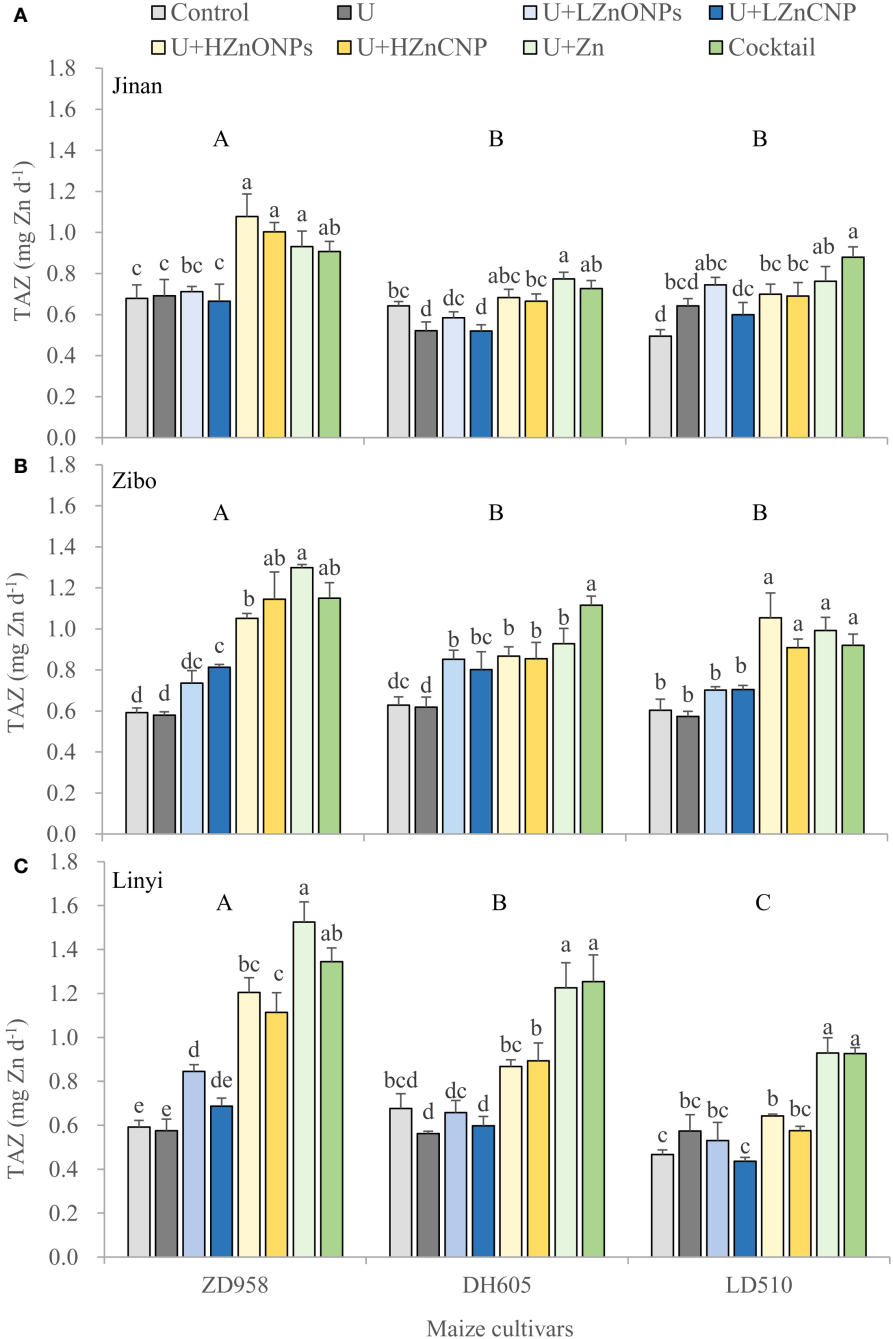
Effects of foliar treatments on total daily absorbed Zn (TAZ) of three maize cultivars grown in Jinan **(A)**, Zibo **(B)** and Linyi **(C)**. Error bars represent the standard error of the mean (n = 3). Bars with different lowercase letters represent significant differences among the different foliar treatments within each maize cultivar; bars with different uppercase letters represent significant differences between different maize cultivars (*P* < 0.05). Control: deionized water; U: urea alone; U+LZnONPs: urea plus ZnO-NPs at low rate; U+LZnCNP: urea plus Zn-CNPs at low rate; U+HZnONPs: urea plus ZnO-NPs at high rate; U+HZnCNP: urea plus Zn-CNPs at high rate; U+Zn: urea plus ZnSO_4_·7H_2_O; Cocktail: mixture of urea, ZnSO_4_·7H_2_O, FeSO_4_·7H_2_O and Na_2_SeO_3_.

**Figure 5:**
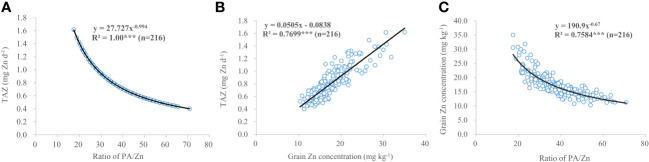
Correlations between ratio of PA/Zn **(A)**, grain Zn concentration **(B)** and TAZ as well as ratio of PA/Zn and grain Zn concentration **(C)** of three maize cultivars grown at three experimental locations. *** indicate correlation coefficient significantly different at *P* < 0.001.

Irrespective of locations and maize cultivars, compared with the control, treatments U, U+LZnONPs and U+LZnCNP did not affect or slightly increased the TAZ while the latter four foliar treatments with high Zn spray rates (especially for treatments U+Zn and cocktail) generally increased the TAZ significantly (**Figure 4**). Due to the fact that TAZ could be well predicted by a negative power function based on the ratios of PA/Zn in grain (R^2 = ^1.00***) (**Figure 5A**), different maize cultivars, experimental locations and foliar treatments had opposite effects on TAZ and the ratios of PA/Zn (**Table 4**). Furthermore, TAZ could be predicted by a linear equation based on grain Zn concentration (R^2 = ^0.7699***) (**Figure 5B**). The relationship between grain Zn concentration and the ratios of PA/Zn in grain could also be described by a negative power function (R^2 = ^0.7584***) (**Figure 5C**).

### Relationships between grain yield and grain nutrient concentrations

**Figure 6:**
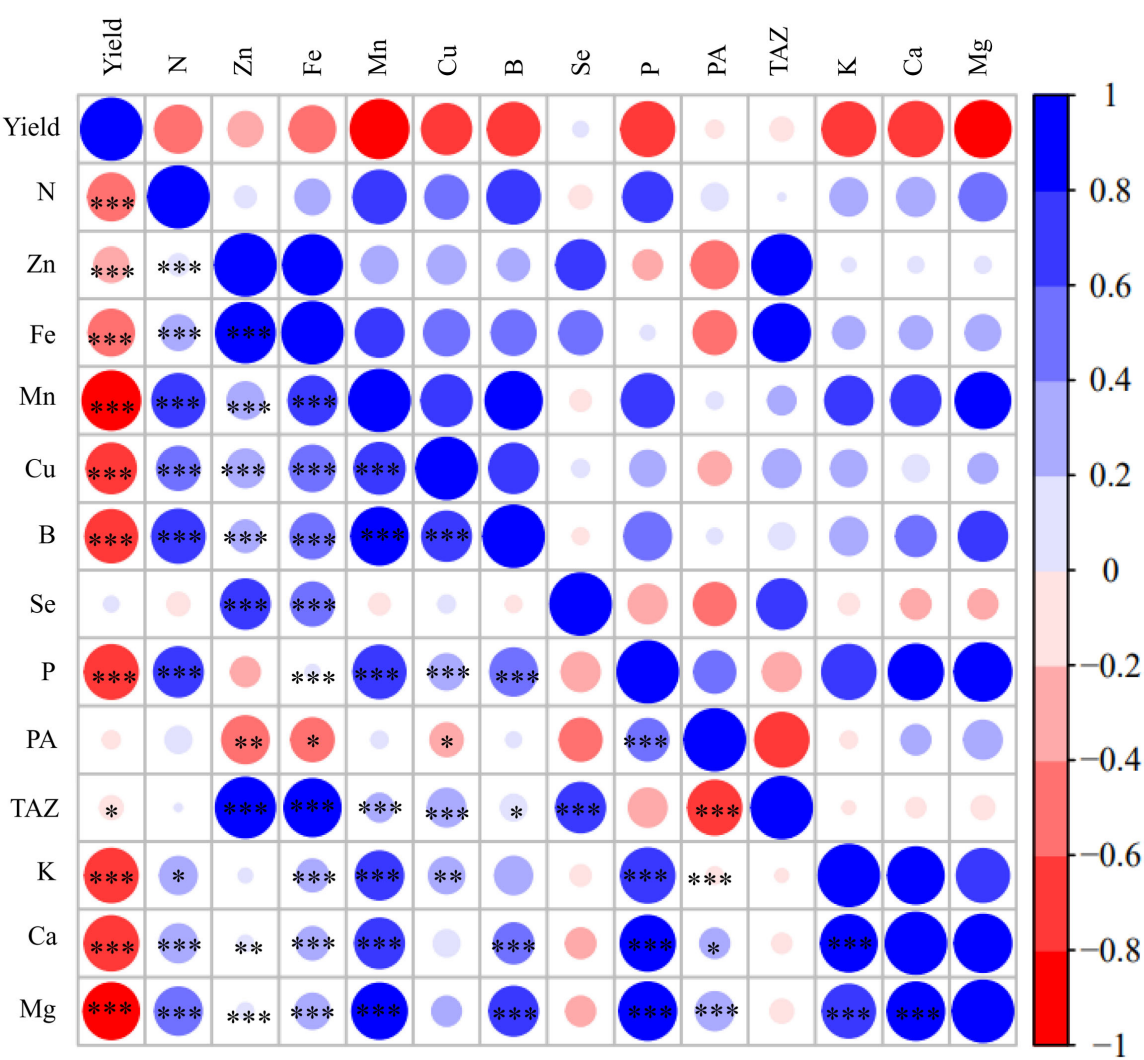
Corrplot representing correlations among measured grain yield and grain concentrations of N, Zn, Fe, Mn, Cu, B, Se, P, phytate acid (PA), TAZ, K, Ca, Mg across different foliar treatments, maize cultivars and experimental locations (n=216). Positive correlations are displayed in red and negative correlations are displayed in blue. The color legend on the right-hand side of corrplot shows correlation coefficients and the corresponding colors. The intensity of the color and the circle size are proportional to the correlation coefficients. *, ** and *** indicate significant correlations at *P* < 0.05, *P* < 0.01 and *P* < 0.001, respectively.

**Figure 6** shows a graphical display of the correlation matrix by corrplot. Grain yield was significantly negatively correlated with all most of grain nutrient concentrations with the correlation coefficient (r values) decreasing in the order Mn, Mg, K, Ca, Cu, P, B, Fe, Zn, N and TAZ. Grain yield was not correlated with the grain Se and PA concentrations. Grain concentrations of N, Zn, Fe, Mn, Cu, B, Ca and Mg have significant positive correlations with each other with the correlation coefficient being the highest between grain concentrations of Zn and Fe (r=0.849***) except that there was no significant correlation between grain Cu concentration and grain Ca and Mg concentrations. In addition, grain Se concentration was significantly positively correlated with grain concentrations of Zn (r=0.461***), Fe (r=0.398***) and TAZ (r=0.403***).

### Principal component analysis of various parameters of maize as affected by experimental locations, cultivars and foliar applications

**Figure 7:**
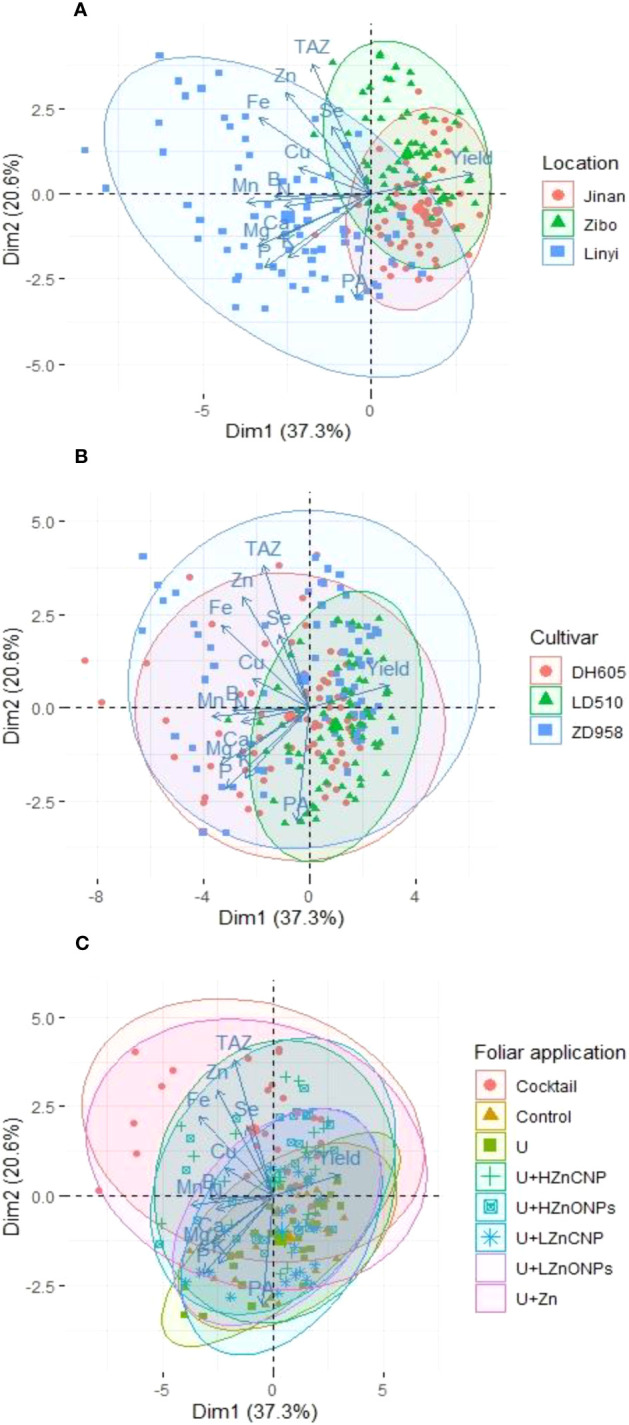
Principal component analysis (PCA) of the effects of different experimental locations **(A)**, maize cultivars **(B)** and foliar treatments **(C)** on various investigated grain yield and nutritional parameters in maize grain (grain concentrations of N, Zn, Fe, Mn, Cu, B, Se, P, PA, TAZ, K, Ca and Mg). Control: deionized water; U: urea alone; U+LZnONPs: urea plus ZnO-NPs at low rate; U+LZnCNP: urea plus Zn-CNPs at low rate; U+HZnONPs: urea plus ZnO-NPs at high rate; U+HZnCNP: urea plus Zn-CNPs at high rate; U+Zn: urea plus ZnSO_4_·7H_2_O; Cocktail: mixture of urea, ZnSO_4_·7H_2_O, FeSO_4_·7H_2_O and Na_₂_SeO_3_.

The PCA showed a better visualization of the relationships and great variation present among all investigated parameters of maize, and it also revealed the data distribution of various treatments performed on the maize crop (**Figure 7**). Two principal components accounted for 57.9% (PC1–37.3%, PC2–20.6%) of the total variance of all 216 data points, which clustered according to experimental locations (**Figure 7A**), maize cultivars (**Figure 7B**) and foliar applications (**Figure 7C**), respectively. There was a clear separation/difference in data distribution areas among three experimental locations and among eight foliar treatments. However, there was much overlap of data distribution areas among the maize cultivars. In addition, in each PCA biplot graph, bigger ellipses indicate less concentrated data and more variation within a cluster. The experimental location in Linyi (**Figure 7A**), maize cultivar of ZD958 (**Figure 7B**) and foliar treatments U+Zn or cocktail (**Figure 7C**) showed the biggest ellipse.

## Discussion

In this study, maize grain yield was first significantly affected by experimental locations, followed by maize cultivars, but not affected by foliar treatments (**Figures 1A–C**, **Supplementary Table 1**). This observation agrees with other studies about wheat, rice and maize (Zhang et al., 2010; Wei et al., 2012; Xia et al., 2019; Zou et al., 2019; Stewart et al., 2020; Wang et al., 2020; Naeem et al., 2022). The non-significant effects of foliar treatments on maize yield might be due to the relatively high concentrations of available Zn and Fe in the three experimental soils (**Table 1**), which were higher than the critical levels in China (0.5 mg kg^–1^ for DTPA-extractable Zn and 5 mg kg^–1^ for DTPA-extractable Fe). Grain yield was significantly negatively correlated with all most of grain nutrient concentrations, with the exception of grain concentrations of Se and PA (**Figure 6**). These results suggest that maize grain nutritional quality tends to decrease with increasing grain yield, especially for high-yielding cultivars, possibly due to the so-called “dilution effect”. Previous studies also demonstrated that maize grain yield was generally negatively correlated with grain concentrations of Fe and Zn as a result of increased carbohydrate content in high-yielding genotypes, which dilutes a given concentration of Fe and Zn (Banziger and Long, 2000; Brkić et al., 2004). Guo et al. (2020) also reported that maize grain yield increased with the year of cultivar release, while the grain concentrations of N, Cu, Mn and Zn significantly declined in the new-era cultivars. Similar results were also found in wheat (Fan et al., 2008). In this study, the significantly higher concentrations of N, Zn, Fe, Se, Mn, B, K, Mg and Ca in grain of maize grown in Linyi than the other two experimental locations were most likely attributed to the lowest grain yield (**Table 3**, **Figures 1**, **3**). Irrespective of experimental locations, the LD510 variety had higher grain yield and biomass compared with ZD958 and DH605, but had the lowest grain concentrations of Zn, Fe, Se, Mn, B and Mg (**Figures 1**, **3**, **Table 3**, **Table S3**). The excessive stay-green LD510 variety may impair the remobilization of these nutrients from vegetative organs to grain, compared with earlier-senescing hybrid ZD958, during the grain filling stage (**Figure S1**), thereafter reducing the grain concentrations of these nutrients. Chen et al. (2016) reported that increasing the translocation of nutrients from vegetative organs to grain could effectively increase the maize grain concentrations of nutrients such as N, P, K, Zn and Mn.

We observed a highly significant positive correlation between maize grain concentrations of Zn and Fe (r=0.849 ***), in agreement with other studies (Chakraborti et al., 2009; Wang et al., 2012; Xia et al., 2019; Zhao et al., 2020), indicating possible synergies between these traits. Co-localization of QTLs for Fe and Zn concentrations revealed that concentration of these minerals could be improved simultaneously by targeting the same chromosomal regions through marker-assisted selection (Qin et al., 2012). Therefore, it is understandable that the U+Zn treatment improved grain concentrations of Zn, Fe and N equally effectively as a cocktail treatment (**Table 3**, **Figure S2**). We also found that foliar application of the cocktail solution was surprisingly effective in increasing maize grain Se concentration, on average by 18-fold (**Table 3**), which is much more pronounced than increases observed in rice (around 4-fold) and wheat (about 3.8-fold) (Zou et al., 2019; Prom-u-thai et al., 2020). Furthermore, grain concentrations of Se were signficantly and positively correlated with Zn and Fe (**Figure 6**). These results suggest that foliar application of a cocktail solution represents an effective strategy to biofortify maize simultaneously with Zn, Se and partly with Fe and N without yield trade-off in maize.

Some researchers (Pfeiffer and McClafferty, 2007; Cakmak et al., 2010) have stated that grain Zn concentrations should be increased by at least 10 mg kg^–1^ to achieve a measurable biological influence on human health. In this present study, the grain Zn concentration of ZD958, DH605 and LD510 grown in Linyi was increased by 17.6 mg kg^–1^, 10.8 mg kg^–1^ and 8.7 mg kg^–1^ by the ZnSO_4_ and micronutrient cocktail treatments (on average) compared with the control treatment, respectively. Thus, the increase in the grain Zn concentration by foliar applications of ZnSO_4_ or a micronutrient cocktail should be sufficient to have a measurable influence on human health. However, the biofortified grain concentrations of Zn and Fe (with a mean of 31.2 mg Zn kg^–1^ and 32.5 mg Fe kg^–1^ of ZD958 grown in Linyi) still could not fulfill the dietary requirements of 38 mg Zn kg^–1^ and 45–60 mg Fe kg^–1^ recommended by HarvestPlus program (Bouis et al., 2011) and therefore, further work is required in this regard. Previous studies revealed a negative effect of foliar application of Zn (with and without urea) on PA concentration in wheat grain and flour (Li et al., 2015). Recent studies also indicated that although the application of ZnO-NPs increased the total Zn within the grain, it did not accumulate within the grain as ZnO-NPs (an important consideration for food safety), but rather mainly as Zn-phytate, with the remainder of the Zn complexed with either cysteine or phosphate (Zhang et al., 2018; Sun et al., 2023). PA, an antinutritional compounds, was significantly decreased by foliar treatements of ZnSO_4_ and the micronutrient cocktail and resulted in the lowest ratios of PA/Fe and PA/Zn and the highest TAZ in maize grain (**Table 4**, **Figure 4**), representing the highest Fe and Zn bioavailability observed in this study. Furthermore, the TAZ could be well estimated by a negative power function based on the ratios of PA/Zn in maize grain (**Figure 5A**).

The effects of different Zn forms and doses on the grain Zn concentration were also investigated in this present study. Results indicates that there was no increase or a negligible increase in grain Zn concentration when 10-fold less Zn (equivalent to 45.2 mg Zn kg^–1^) was foliar applied as nano-fertilizers, compared with the control (deionized water) (**Figures 3A-C**). In contrast, higher rates of foliar-applied Zn forms resulted in a significant increase in grain Zn concentration with the positive effects being dependent on experimental locations and maize cultivars. In case of the Linyi experiment, the magnitude of increase in grain Zn concentration revealed the greater efficacy of conventional ZnSO_4_ than either ZnO-NPs or Zn-CNPs (**Figures 3A-C**). Similarly, Doolette et al. (2020) reported that conventional formulations such as ZnCl_2_ and especially Zn-EDTA were more effective in terms of the proportion of applied ^65^Zn translocated to grain compared with ZnO-NPs and microparticles by applying ^65^Zn radio-labelled formulations onto the youngest fully emerged leaf of wheat. In disagreement with these results, Zhang et al. (2018) reported the improvement in grain Zn concentration of wheat was more pronounced by foliar-applied ZnO NPs than conventional ZnSO_4_ based on the same level of Zn. It is possible that ZnO-NPs or Zn-CNPs provide benefits beyond simply increasing grain micronutrient concentrations. For example, Choudhary et al. (2019) found that Zn-chitosan nanoparticles exhibited significant disease control through strengthening of plant innate immunity by elevating antioxidant and defense enzymes, balancing of reactive oxygen species, and enhancing lignin accumulation. Furthermore, seed treatment together with foliar application of Zn-chitosan nanoparticles at rates of 0.01–0.16% significantly controlled Curvularia leaf spot disease and increased grain yield from 20.5% to 39.8% alongside an improvement in grain Zn concentration from 41.3 to 62.2 mg kg^–1^ (Choudhary et al., 2019). The differences between different Zn sources, different cultivars, and different experimental locations on grain Zn biofortification could be explained by a number of factors. First, plant physiology between different crop species and cultivars may affect Zn demand and cuticle properties (e.g. cuticle thickness, and stomata and trichome density). Second, the difference in nanoparticle particle size may affect the adhesion and rate of Zn uptake by the leaf. Third, the different concentrations and dosages of Zn for foliar application may affect Zn uptake by the leaf. In our study we applied 45.2 or 452 mg Zn kg^–1^ but rates of 1600 mg Zn kg^–1^ have been applied by others (Zhang et al., 2018). Finally, the growing conditions (field vs solution culture and pot experiments) and yield levels may also affect the Zn uptake and allocation to grain.

The maize cultivars DH605 and LD510 used in this study are intended for dual use of grain and silage. The maize is usually harvested by farmers as grain or silage based on local maize purchase prices and silage corn is often purchased by large dairy farmers/farm. Previous studies report the recommended dietary requirements of minerals (based on dry weight) for dairy cows (**Supplementary Table 4**). Considering the requirements of dairy cattle for Mn and Fe, the average shoot concentrations of the two maize cultivars across the three locations were sufficient to meet Mn (12–14 mg kg^–1^) and Fe (12.3–18 mg kg^–1^) requirements with or without foliar treatments. However, the average shoot concentrations of Cu, K, Mg and Ca could not easily meet the dietary requirements of dairy cattle, irrespective of foliar treatments. The shoot Se concentration achieved 765.2 ug kg^–1^ for DH605 and 562.1 ug kg^–1^ for LD510 with foliar treatment cocktail, which indicated that Se biofortified silage could easily meet the requirement of 300 ug Se kg^–1^ for dairy cattle (**Supplementary Table 4**). The shoot Zn concentration of the two maize cultivars ranged between 51.6–66.6 mg kg^–1^ when treated with the ZnSO_4_ and micronutrient cocktail foliar treatments. This indicates that the Zn biofortified silage could meet the requirements of 43–60 mg Zn kg^–1^ for dairy cattle. Furthermore, the ratios of P/Zn and P/Fe in shoot and straw were also reduced by foliar treatments ZnSO_4_ and the micronutrient cocktail (**Table 4**), indicating a higher bioavailability of Zn and Fe for dairy cattle by biofortification.

## Conclusion

Results from this present study have clearly demonstrated that grain yield was first significantly affected by the experimental locations, followed by maize cultivars, but not affected by foliar treatments. Additionally, grain yield was significantly negatively correlated with all most of grain nutrient concentrations with an exception of grain concentrations of Se and PA. Consequently, the high grain yield of cultivar LD510 or high grain yield in Jinan and Zibo nonorally resulted in the lower grain nutritional quality possibly due to “dilution effect”. Therefore, foliar fertilizer sprays should be preferentially applied under these conditions to improve grain nutritional quality while maintaining higher grain yield. It was shown that foliar application of a cocktail solution including Zn, Fe, Se and N simultaneously has effectively improved the concentrations of Zn, Fe, Se and N in grain of the three maize cultivars grown at three locations without grain yield trade-off. Furthermore, foliar application of a cocktail solution also significantly decreased the PA concentration, the ratios of PA/Fe and PA/Zn in grains while increased the TAZ, indicating an increased Fe and Zn bioavailability. Among the different Zn sources, foliar application of conventional ZnSO_4_·7H_2_O was more pronounced in improve the grain Zn concentration of maize than that of ZnO-NPs and Zn-CNPs in this study and the relevant mechanisms need to be further investigated in the future. It is also important to mention that the ongoing breeding activities for developing high-yield genotypes should be integrated with agronomic approaches (e.g. foliar application of a cocktail solution including Zn, Fe, Se and N simultaneously) to achieve both high grain yield and high grain nutritional quality for human health.

## Data availability statement

The original contributions presented in the study are included in the article/**Supplementary material**. Further inquiries can be directed to the corresponding authors.

## Author contributions

Y-FX and Z-LC conceived and designed the experiments. X-JL, WY, QM, C-YZ, MH, J-BS, S-JQ, Z-HD performed the experiments. Y-FX and Z-LC analyzed the data and wrote the paper. All the authors read and approved the final manuscript.

## Supplementary material

Supplementary Figure 1 The growth of three maize cultivars at 45 days after silking grown in Jinan.

Supplementary Figure 2 Effects of foliar treatments on Fe concentrations of grain (A-C) and straw (D-F) of different maize cultivars grown in Jinan, Zibo and Linyi, respectively. Error bars represent the standard error of the mean (n = 3). Bars with different lowercase letters represent significant differences among the different foliar treatments within each maize cultivar; bars with different uppercase letters represent significant differences between different maize cultivars (*P* < 0.05). Control: deionized water; U: urea alone; U+LZnONPs: urea plus ZnO-NPs at low rate; U+LZnCNP: urea plus Zn-CNPs at low rate; U+HZnONPs: urea plus ZnO-NPs at high rate; U+HZnCNP: urea plus Zn-CNPs at high rate; U+Zn: urea plus ZnSO_4_·7H_2_O; Cocktail: mixture of urea, ZnSO_4_·7H_2_O, FeSO_4_·7H_2_O and Na_2_SeO_3_.

Supplementary Figure 3 Effects of foliar treatments on Se concentrations of grain (A-C) and straw (D-F) of different maize cultivars grown in Jinan, Zibo and Linyi, respectively. Error bars represent the standard error of the mean (n = 3). Bars with different lowercase letters represent significant differences among the different foliar treatments within each maize cultivar; bars with different uppercase letters represent significant differences between different maize cultivars (*P* < 0.05). Control: deionized water; U: urea alone; U+LZnONPs: urea plus ZnO-NPs at low rate; U+LZnCNP: urea plus Zn-CNPs at low rate; U+HZnONPs: urea plus ZnO-NPs at high rate; U+HZnCNP: urea plus Zn-CNPs at high rate; U+Zn: urea plus ZnSO_4_·7H_2_O; Cocktail: mixture of urea, ZnSO_4_·7H_2_O, FeSO_4_·7H_2_O and Na_2_SeO_3_.

Supplementary Figure 4 Effects of foliar treatments on grain N concentrations of different maize cultivars grown in Jinan (A), Zibo (B) and Linyi (C). Error bars represent the standard error of the mean (n = 3). Bars with different lowercase letters represent significant differences among the different foliar treatments within each maize cultivar; bars with different uppercase letters represent significant differences between different maize cultivars (*P* < 0.05). Control: deionized water; U: urea alone; U+LZnONPs: urea plus ZnO-NPs at low rate; U+LZnCNP: urea plus Zn-CNPs at low rate; U+HZnONPs: urea plus ZnO-NPs at high rate; U+HZnCNP: urea plus Zn-CNPs at high rate; U+Zn: urea plus ZnSO_4_·7H_2_O; Cocktail: mixture of urea, ZnSO_4_·7H_2_O, FeSO_4_·7H_2_O and Na_2_SeO_3_.
