# Supplementary material for: Biofortification of different maize cultivars with zinc, iron and selenium by foliar fertilizer applications
Source: Front Plant Sci. 2023 Sep 7;14:1144514. doi: 10.3389/fpls.2023.1144514 (PMC10513412; doi:10.3389/fpls.2023.1144514)
Supplement: Supplementary file 1 [file DataSheet_1.docx]

**Supplementary materials**

**TABLE S1** Three-way analysis of variance (ANOVA) of the effects of experimental locations, maize cultivars, foliar treatments and their double and triple interactions on the grain yield, biomass, grain Zn concentration (GZnC)_,_ straw Zn concentration (SZnC) and total daily absorbed Zn (TAZ)

| Treatment | DF | Yield | |  | Biomass | |  | GZnC | |  | SZnC | |  | TAZ | |
| --- | --- | --- | --- | --- | --- | --- | --- | --- | --- | --- | --- | --- | --- | --- | --- |
|  |  | SS | F Pr |  | SS | F Pr |  | SS | F Pr |  | SS | F Pr |  | SS | F Pr |
| Location (L) | 2 | 186.28 | <0.0001 |  | 516.39 | <0.0001 |  | 150.70 | <0.0001 |  | 2993.71 | <0.0001 |  | 0.69 | <0.0001 |
| Cultivar (C) | 2 | 32.14 | <0.0001 |  | 67.95 | <0.0001 |  | 574.93 | <0.0001 |  | 256.67 | ns |  | 1.56 | <0.0001 |
| Foliar application (F) | 7 | 2.79 | ns |  | 10.88 | ns |  | 2074.52 | <0.0001 |  | 290395.23 | <0.0001 |  | 6.49 | <0.0001 |
| L*C | 4 | 4.15 | ns |  | 24.07 | 0.0099 |  | 238.07 | <0.0001 |  | 3623.24 | 0.0003 |  | 0.59 | <0.0001 |
| L*F | 14 | 5.14 | ns |  | 9.15 | ns |  | 436.81 | <0.0001 |  | 25806.60 | <0.0001 |  | 1.14 | <0.0001 |
| C*F | 14 | 7.47 | ns |  | 23.51 | ns |  | 154.44 | <0.0001 |  | 5213.73 | 0.0047 |  | 0.76 | <0.0001 |
| L*C*F | 28 | 15.29 | ns |  | 55.2 | ns |  | 116.22 | 0.0008 |  | 8472.81 | 0.0023 |  | 0.55 | 0.0082 |

ns indicates nonsignificance.

**TABLE S2** Two-way analysis of variance (ANOVA) of the effects of maize cultivars, foliar treatments (Control, U+HZnONPs, U+HZnCNP and U+Zn ) and their interactions on the leaf *P_n_*, *G_s,_ T_r_* and *C_i_* at 30 and 45 days after silking (DAS) in Jinan

| Source of variation | DF | 30 DAS | | | | | | | | | | |
| --- | --- | --- | --- | --- | --- | --- | --- | --- | --- | --- | --- | --- |
|  |  | Pn | |  | Gs | |  | Tr | |  | Ci | |
|  |  | SS | F Pr. |  | SS | F Pr. |  | SS | F Pr. |  | SS | F Pr. |
| Cultivar (C) | 2 | 146 | 0.0005 |  | 0.1631 | <0.0001 |  | 60.97 | <0.0001 |  | 58616.3 | <0.0001 |
| Foliar application (F) | 3 | 9.24 | ns |  | 0.0073 | ns |  | 2.45 | ns |  | 1259.47 | ns |
| C*F | 6 | 16.57 | ns |  | 0.011 | ns |  | 1.28 | ns |  | 785.44 | ns |
| Source of variation | DF | 45 DAS | | | | | | | | | | |
|  |  | Pn | |  | Gs | |  | Tr | |  | Ci | |
|  |  | SS | F Pr. |  | SS | F Pr. |  | SS | F Pr. |  | SS | F Pr. |
| Cultivar (C) | 2 | 512.01 | <0.0001 |  | 0.0411 | <0.0001 |  | 23.79 | 0.0003 |  | 3334.59 | 0.0454 |
| Foliar application (F) | 3 | 93.58 | ns |  | 0.0022 | ns |  | 3.45 | ns |  | 4118.15 | ns |
| C*F | 6 | 66.54 | ns |  | 0.015 | ns |  | 11.51 | ns |  | 2794.27 | ns |

ns indicates nonsignificance.

Control: deionized water; U+HZnONPs: urea plus ZnO-NPs at high rate; U+HZnCNP: urea plus Zn-CNPs at high rate; U+Zn: urea plus ZnSO_4_·7H_2_O

**TABLE S3** Effects of foliar treatments on Mn, Cu, B, K, Mg and Ca concentrations in grain of three maize cultivars grown at three experimental locations

| Treatment | Mn | Cu | B | K | Mg | Ca |
| --- | --- | --- | --- | --- | --- | --- |
|  | (mg kg^–1^) | (mg kg^–1^) | (mg kg^–1^) | (g kg^–1^) | (g kg^–1^) | (g kg^–1^) |
| Location (L) |  |  |  |  |  |  |
| Jinan | 2.59c | 0.64b | 1.42c | 3.88b | 0.89b | 0.05b |
| Zibo | 2.93b | 1.55a | 1.86b | 3.41c | 0.80c | 0.03c |
| Linyi | 3.68a | 1.60a | 2.22a | 4.56a | 0.99a | 0.07a |
| Cultivar (C) |  |  |  |  |  |  |
| ZD958 | 3.21a | 1.32a | 1.64b | 4.06a | 0.89b | 0.05a |
| DH605 | 3.29a | 1.18b | 2.41a | 3.77b | 0.94a | 0.05a |
| LD510 | 2.70b | 1.30a | 1.44c | 4.03a | 0.86c | 0.05a |
| Foliar application (F) |  |  |  |  |  |  |
| Control | 3.08a | 1.25a | 1.85a | 3.94a | 0.90a | 0.05a |
| U | 3.09a | 1.32a | 1.84a | 4.00a | 0.89a | 0.05a |
| U+LZnONPs | 3.14a | 1.21a | 1.82a | 4.01a | 0.90a | 0.04a |
| U+LZnCNP | 3.00a | 1.25a | 1.76a | 4.06a | 0.90a | 0.05a |
| U+HZnONPs | 3.02a | 1.29a | 1.81a | 3.88a | 0.88a | 0.04a |
| U+HZnCNP | 3.03a | 1.25a | 1.83a | 3.89a | 0.90a | 0.05a |
| U+Zn | 3.21a | 1.29a | 1.93a | 3.89a | 0.90a | 0.05a |
| Cocktail | 2.97a | 1.26a | 1.81a | 3.95a | 0.91a | 0.05a |
| Source of variation |  |  |  |  |  |  |
| L | <0.0001 | <0.0001 | <0.0001 | <0.0001 | <0.0001 | <0.0001 |
| C | <0.0001 | <0.0001 | <0.0001 | <0.0001 | <0.0001 | ns |
| F | ns | ns | ns | ns | ns | ns |
| L*C | <0.0001 | <0.0001 | <0.0001 | <0.0001 | <0.0001 | <0.0001 |
| L*F | ns | ns | 0.0027 | ns | ns | 0.011 |
| C*F | ns | ns | ns | ns | ns | ns |
| L*C*F | ns | ns | 0.0025 | 0.0430 | ns | ns |

Means in a column followed by different lowercase letters are significantly different among different locations, cultivars and foliar treatments (*P*<0.05). ns indicates nonsignificance.

Control: deionized water; U: urea alone; U+LZnONPs: urea plus ZnO-NPs at low rate; U+LZnCNP: urea plus Zn-CNPs at low rate; U+HZnONPs: urea plus ZnO-NPs at high rate; U+HZnCNP: urea plus Zn-CNPs at high rate; U+Zn: urea plus ZnSO_4_·7H_2_O; Cocktail: mixture of urea, ZnSO_4_·7H_2_O, FeSO_4_·7H_2_O and Na₂SeO_3_.

**TABLE S4** Zn, Fe, Mn, Cu, Se, P, K, Mg and Ca concentrations in shoot of DH605 and LD510 across the different experimental locations

| Cultivar | Treatment | Zn | Fe | Mn | Cu | Se | P | K | Mg | Ca |  |
| --- | --- | --- | --- | --- | --- | --- | --- | --- | --- | --- | --- |
|  |  | (mg kg^–1^) | (mg kg^–1^) | (mg kg^–1^) | (mg kg^–1^) | (ug kg^–1^) | (g kg^–1^) | (g kg^–1^) | (g kg^–1^) | (g kg^–1^) |  |
| DH605 | Control | 16.02 | 84.55 | 14.46 | 2.86 | 54.8 | 1.89 | 6.2 | 1.57 | 1.9 |  |
|  | U | 15.03 | 72.01 | 15.16 | 2.98 | 55.04 | 1.82 | 6.28 | 1.41 | 1.82 |  |
|  | U+LZnONPs | 18.51 | 75.83 | 13.78 | 2.88 | 53.12 | 1.8 | 7.32 | 1.37 | 1.66 |  |
|  | U+LZnCNP | 16.98 | 84.46 | 15.62 | 2.97 | 58.43 | 1.78 | 7.52 | 1.39 | 1.89 |  |
|  | U+HZnONPs | 32.49 | 85.96 | 14.69 | 2.87 | 57.57 | 1.75 | 6.85 | 1.47 | 1.82 |  |
|  | U+HZnCNP | 40.35 | 85.95 | 14.43 | 2.96 | 64.02 | 1.73 | 7.95 | 1.46 | 1.9 |  |
|  | U+Zn | 66.6 | 84.79 | 15 | 2.84 | 59.08 | 1.72 | 6.78 | 1.45 | 1.86 |  |
|  | Cocktail | 54.14 | 122.43 | 12.69 | 2.54 | 765.22 | 1.75 | 7.51 | 1.34 | 1.68 |  |
| LD510 | Control | 12.45 | 59.77 | 19.18 | 2.72 | 56.15 | 1.91 | 7.77 | 1.53 | 1.94 |  |
|  | U | 13.25 | 70.21 | 19.65 | 2.68 | 43.44 | 1.77 | 7.32 | 1.56 | 2 |  |
|  | U+LZnONPs | 18.43 | 59.1 | 16.7 | 2.69 | 44.15 | 1.81 | 7.78 | 1.55 | 2.07 |  |
|  | U+LZnCNP | 17.31 | 68.74 | 16.61 | 2.81 | 59.57 | 1.83 | 7.82 | 1.64 | 2.06 |  |
|  | U+HZnONPs | 40.47 | 61.71 | 18.98 | 2.59 | 46.61 | 1.78 | 8.47 | 1.5 | 2.08 |  |
|  | U+HZnCNP | 37.16 | 70.02 | 14.76 | 2.5 | 51.65 | 1.74 | 7.93 | 1.57 | 1.93 |  |
|  | U+Zn | 60.22 | 59.94 | 16.86 | 2.62 | 46.68 | 1.75 | 8.23 | 1.48 | 1.98 |  |
|  | Cocktail | 51.56 | 100.82 | 13.21 | 2.47 | 562.11 | 1.71 | 7.99 | 1.53 | 1.92 |  |
| **Recommended value^a^** | | **43–60** | **12.3–18** | **12–14** | **9–11** | **300** | **3.2–4.4** | **10** | **1.8-2.1** | **5.3–6.7** |  |
|  |  |  |  |  |  |  |  |  |  |  |  |

^a^ Recommended dietary requirement of minerals for dairy cows according to National Research Council.

National Research Council. Nutrient requirements of dairy cattle, 7th revised edition, The National Academies Press, Washington, DC, USA, 2001.

Control: deionized water; U: urea alone; U+LZnONPs: urea plus ZnO-NPs at low rate; U+LZnCNP: urea plus Zn-CNPs at low rate; U+HZnONPs: urea plus ZnO-NPs at high rate; U+HZnCNP: urea plus Zn-CNPs at high rate; U+Zn: urea plus ZnSO_4_·7H_2_O; Cocktail: mixture of urea, ZnSO_4_·7H_2_O, FeSO_4_·7H_2_O and Na₂SeO_3_.
