# Supplementary figures and images for: Biofortification of different maize cultivars with zinc, iron and selenium by foliar fertilizer applications
Source: Front Plant Sci. 2023 Sep 7;14:1144514. doi: 10.3389/fpls.2023.1144514 (PMC10513412; doi:10.3389/fpls.2023.1144514)

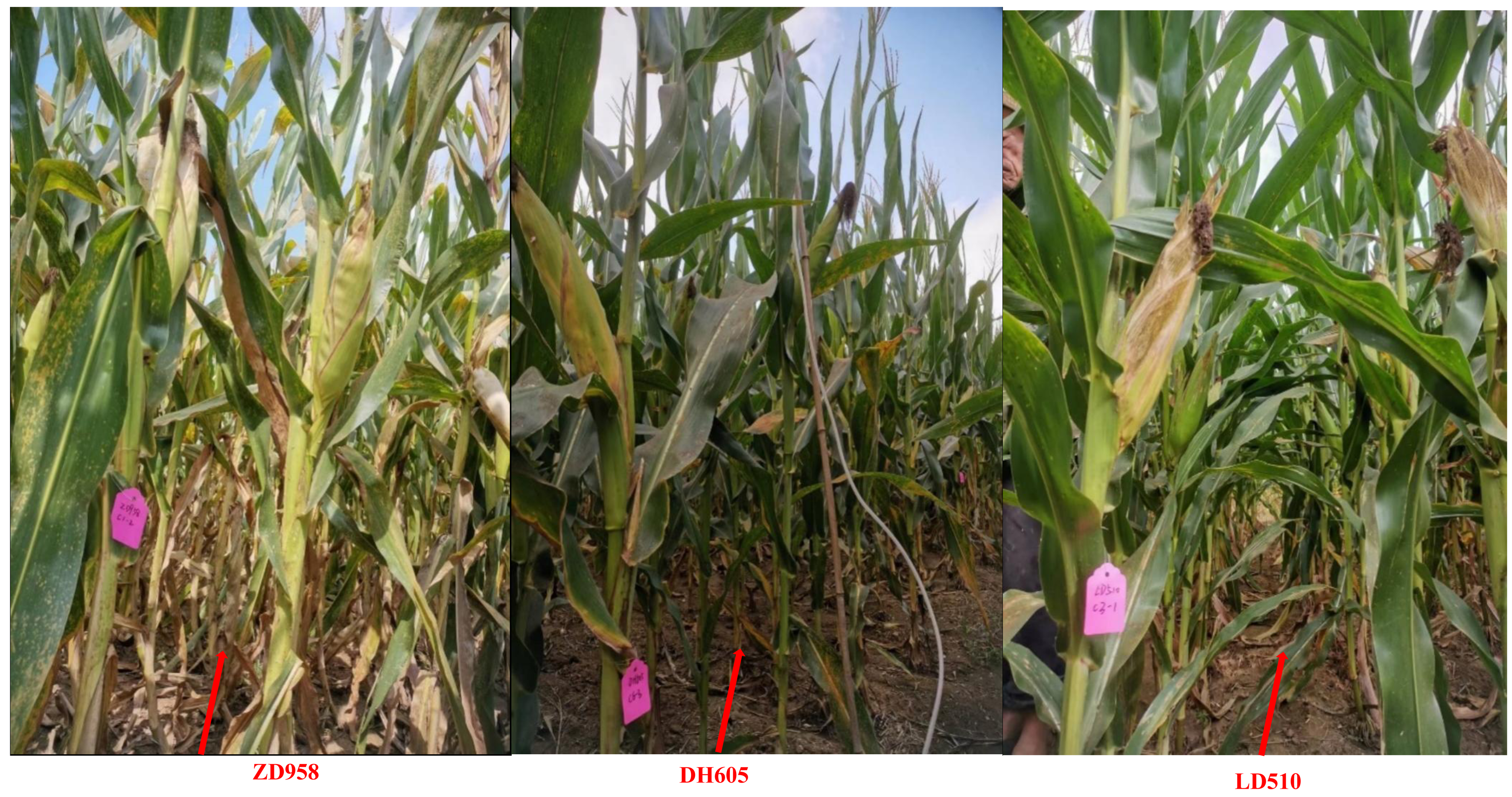

Supplement: Supplementary Figure 1 — The growth of three maize cultivars at 45 days after silking grown in Jinan. [file Image_1.tif]

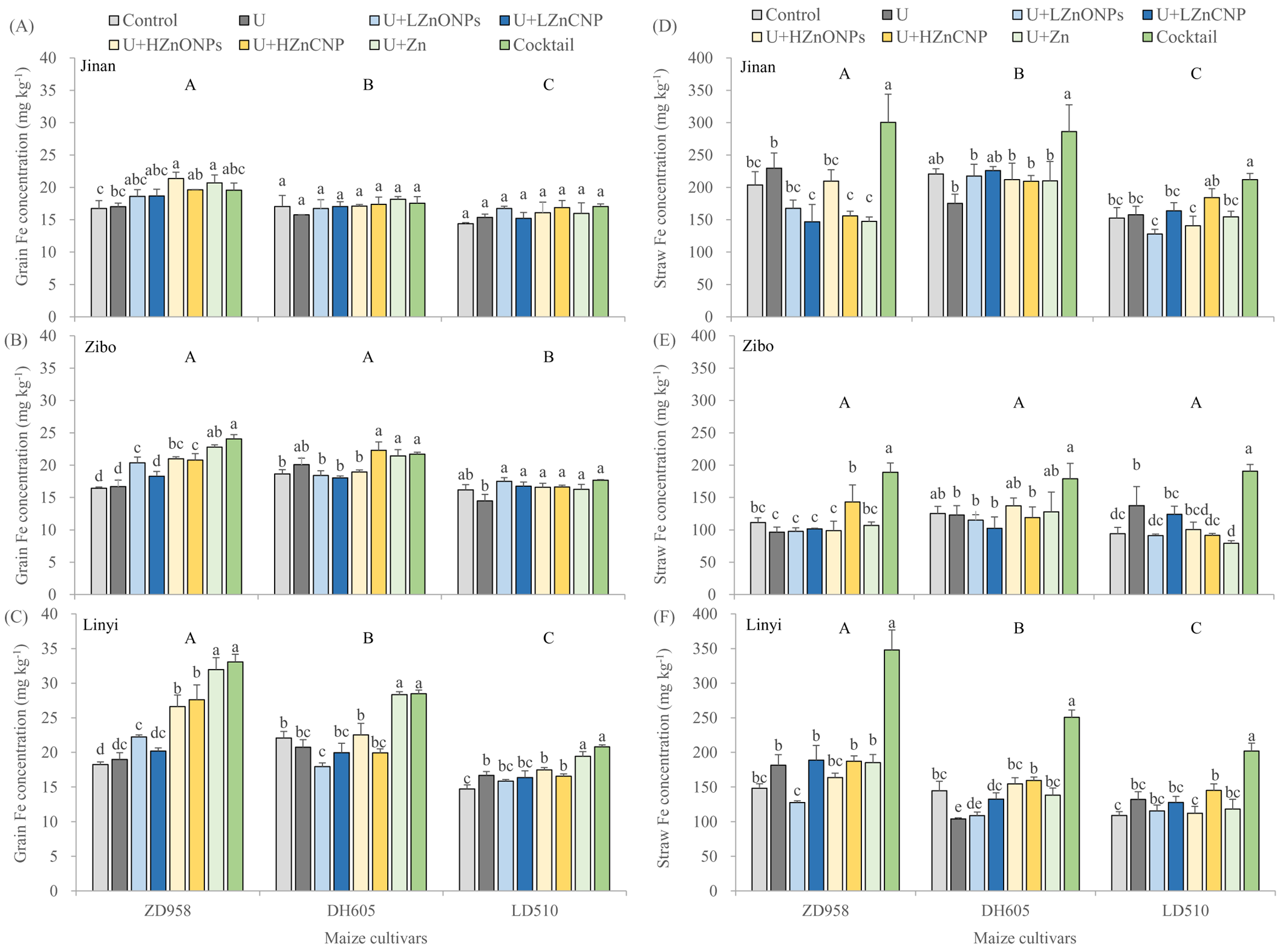

Supplement: Supplementary Figure 2 — Effects of foliar treatments on Fe concentrations of grain (A-C) and straw (D-F) of different maize cultivars grown in Jinan, Zibo and Linyi, respectively. Error bars represent the standard error of the mean (n = 3). Bars with different lowercase letters represent significant differences among the different foliar treatments within each maize cultivar; bars with different uppercase letters represent significant differences between different maize cultivars (P < 0.05). Control: deionized water; U: urea alone; U+LZnONPs: urea plus ZnO-NPs at low rate; U+LZnCNP: urea plus Zn-CNPs at low rate; U+HZnONPs: urea plus ZnO-NPs at high rate; U+HZnCNP: urea plus Zn-CNPs at high rate; U+Zn: urea plus ZnSO4·7H2O; Cocktail: mixture of urea, ZnSO4·7H2O, FeSO4·7H2O and Na2SeO3. [file Image_2.tif]

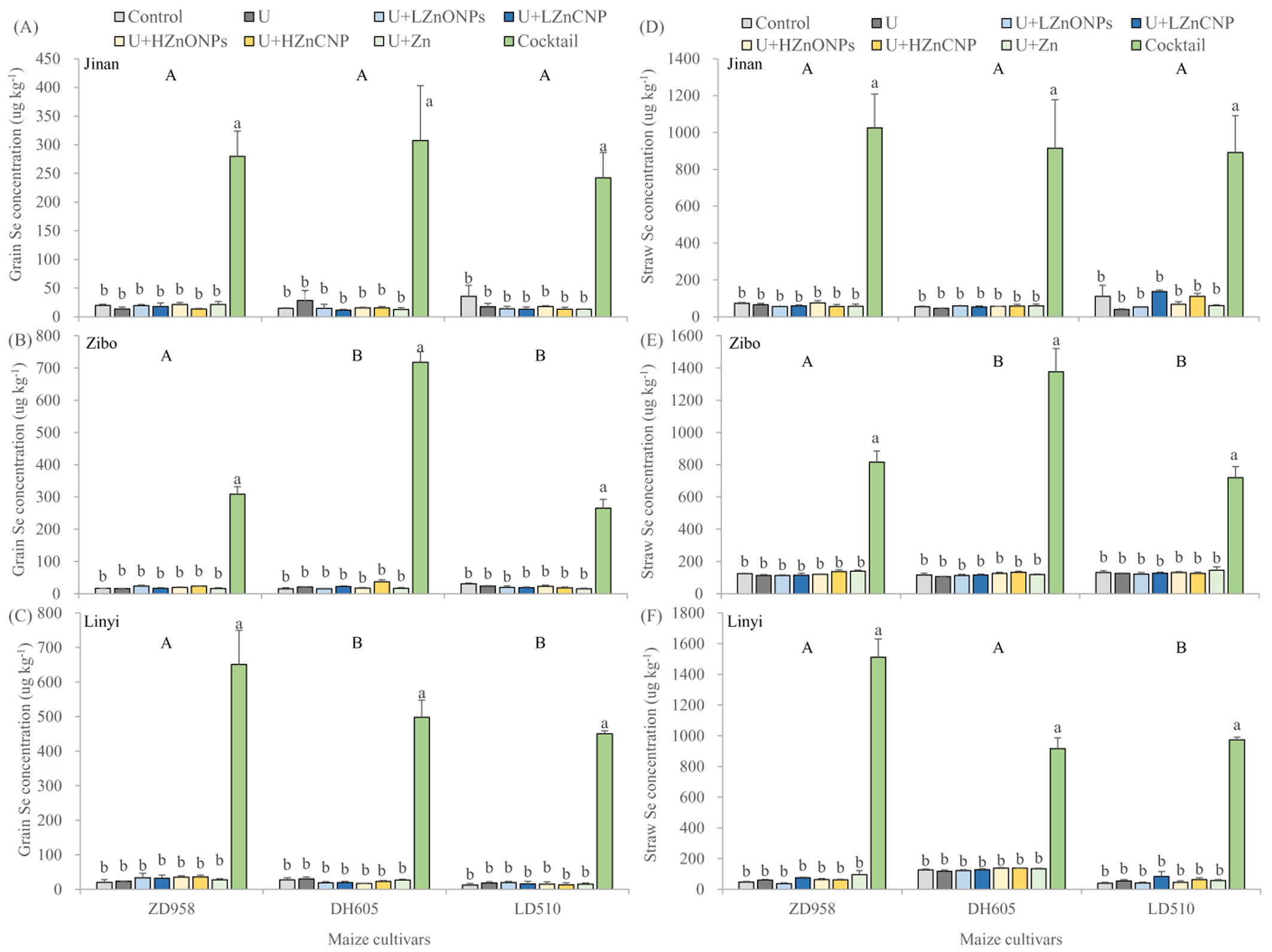

Supplement: Supplementary Figure 3 — Effects of foliar treatments on Se concentrations of grain (A-C) and straw (D-F) of different maize cultivars grown in Jinan, Zibo and Linyi, respectively. Error bars represent the standard error of the mean (n = 3). Bars with different lowercase letters represent significant differences among the different foliar treatments within each maize cultivar; bars with different uppercase letters represent significant differences between different maize cultivars (P < 0.05). Control: deionized water; U: urea alone; U+LZnONPs: urea plus ZnO-NPs at low rate; U+LZnCNP: urea plus Zn-CNPs at low rate; U+HZnONPs: urea plus ZnO-NPs at high rate; U+HZnCNP: urea plus Zn-CNPs at high rate; U+Zn: urea plus ZnSO4·7H2O; Cocktail: mixture of urea, ZnSO4·7H2O, FeSO4·7H2O and Na2SeO3. [file Image_3.tif]

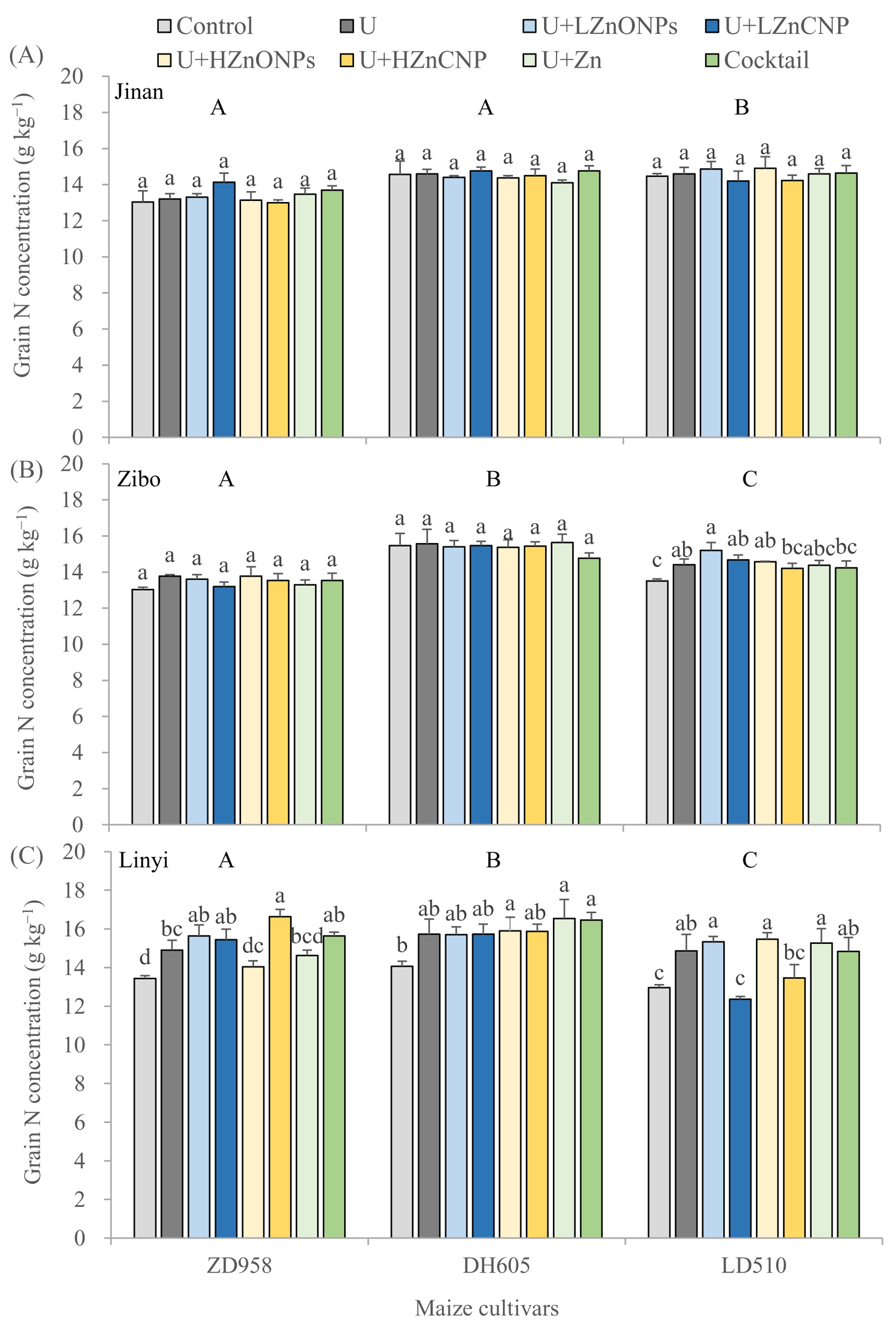

Supplement: Supplementary Figure 4 — Effects of foliar treatments on grain N concentrations of different maize cultivars grown in Jinan (A), Zibo (B) and Linyi (C). Error bars represent the standard error of the mean (n = 3). Bars with different lowercase letters represent significant differences among the different foliar treatments within each maize cultivar; bars with different uppercase letters represent significant differences between different maize cultivars (P < 0.05). Control: deionized water; U: urea alone; U+LZnONPs: urea plus ZnO-NPs at low rate; U+LZnCNP: urea plus Zn-CNPs at low rate; U+HZnONPs: urea plus ZnO-NPs at high rate; U+HZnCNP: urea plus Zn-CNPs at high rate; U+Zn: urea plus ZnSO4·7H2O; Cocktail: mixture of urea, ZnSO4·7H2O, FeSO4·7H2O and Na2SeO3. [file Image_4.tif]
